# Supplementary figures and images for: Metadherin Regulates Inflammatory Breast Cancer Invasion and Metastasis
Source: Int J Mol Sci. 2023 Feb 28;24(5):4694. doi: 10.3390/ijms24054694 (PMC10002532; doi:10.3390/ijms24054694)

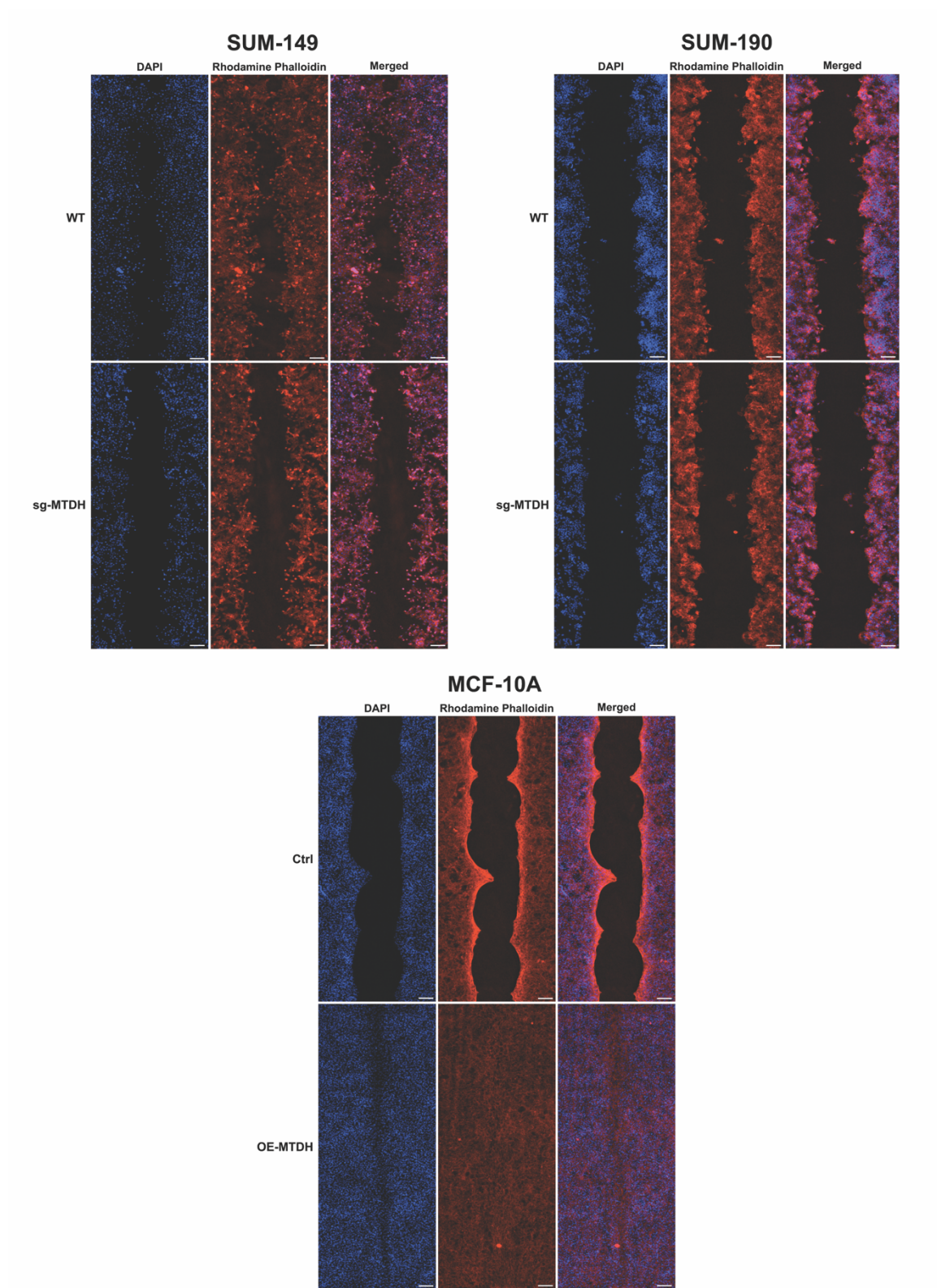

**Figure S1:** Wound healing assay micrographs at a magnification of 4X. Scale bar = 50 μm.

Supplement: Supplementary file 1 [file ijms-24-04694-s001.zip › ijms-2175762-supplementary.pdf]
